# Validation and Characterization of Maize Multiple Disease Resistance QTL

**DOI:** 10.1534/g3.119.400195

**Published:** 2019-07-12

**Authors:** Lais B. Martins, Elizabeth Rucker, Wade Thomason, Randall J. Wisser, James B. Holland, Peter Balint-Kurti

**Affiliations:** * Dept. of Crop Science, North Carolina State University, Box 7620, Raleigh, NC 27695; † School of Plant and Environmental Sciences, Virginia Tech, Blacksburg, VA 24061; ‡ Dept. of Plant and Soil Sciences, University of Delaware, Newark, DE 19716; § Plant Science Research Unit, USDA-ARS, Raleigh NC 27695-7616; ** Dept. of Entomology and Plant Pathology, North Carolina State University, Box 7616 Raleigh, NC 27695

**Keywords:** Disease, Maize, QTL, Resistance, Genetics of Immunity

## Abstract

Southern Leaf Blight, Northern Leaf Blight, and Gray Leaf Spot, caused by ascomycete fungi, are among the most important foliar diseases of maize worldwide. Previously, disease resistance quantitative trait loci (QTL) for all three diseases were identified in a connected set of chromosome segment substitution line (CSSL) populations designed for the identification of disease resistance QTL. Some QTL for different diseases co-localized, indicating the presence of multiple disease resistance (MDR) QTL. The goal of this study was to perform an independent test of several of the MDR QTL identified to confirm their existence and derive a more precise estimate of allele additive and dominance effects. Twelve F_2:3_ family populations were produced, in which selected QTL were segregating in an otherwise uniform genetic background. The populations were assessed for each of the three diseases in replicated trials and genotyped with markers previously associated with disease resistance. Pairwise phenotypic correlations across all the populations for resistance to the three diseases ranged from 0.2 to 0.3 and were all significant at the alpha level of 0.01. Of the 44 QTL tested, 16 were validated (identified at the same genomic location for the same disease or diseases) and several novel QTL/disease associations were found. Two MDR QTL were associated with resistance to all three diseases. This study identifies several potentially important MDR QTL and demonstrates the importance of independently evaluating QTL effects following their initial identification.

Genetic resistance is usually the most cost-effective method of controlling crop disease. Qualitative disease resistance confers high levels of resistance, is typically race-specific and controlled by one or few genes with major effects (Bent and Mackey 2007). By contrast, quantitative disease resistance (QDR) causes a partial reduction in disease symptoms, is usually controlled by multiple genes with relatively small effects and is often not specific to particular pathogen races (Niks *et al.* 2015; Yang *et al.* 2017a). QDR is thought to be more durable than qualitative resistance because it relies on the resistance mechanisms associated with many different genes, each of which exerts a relatively small selection pressure on pathogens (Agrios 2005).

Multiple disease resistance (MDR) loci, defined as “loci that confer resistance to two or more diseases” (Nene 1988; Wiesner-Hanks and Nelson 2016), can be due to distinct linked genes, each associated with resistance to one disease, or to an individual allele that confers resistance to more than one disease (pleiotropy). Colocalization of QTL for resistance to Southern leaf blight (SLB), Northern leaf blight (NLB) and Gray leaf spot (GLS) diseases, caused by the fungi *Cochliobolus heterostrophus*, *Setosphaeria turcica* and *Cercospora zeae-maydis*/*Cercospora zeina*, respectively, has been reported in maize (Balint-Kurti *et al.* 2010; Zwonitzer *et al.* 2010; Wisser *et al.* 2011; Belcher *et al.* 2012; Li *et al.* 2018; Lopez-Zuniga *et al.* 2019). These diseases are among the most important foliar diseases of maize worldwide (Mueller *et al.* 2016). The pathogens are all ascomycete fungi in the dothideomycete class that share a largely necrotrophic lifestyle (although *S. turcica* has been described as a hemi-biotroph). One gene associated with detoxification (Wisser *et al.*, 2011) and another involved in lignin biosynthesis (Yang *et al.* 2017b) appear to confer resistance to all three diseases.

Wisser *et al.* (2011) found high positive genetic correlations between resistance to these diseases in a maize association mapping panel of 253 maize inbred lines (Flint-Garcia *et al.* 2005). Linkage disequilibrium in this population is very low, reducing the chances that the correlations among resistances were due to linkage rather than pleiotropy. Several MDR and multiple disease susceptible (MDS) lines were selected from this population for the development of populations designed for the mapping and characterization of MDR loci. We developed a set of BC_3_F_4:5_ chromosome segment substitution line (CSSL) populations in which segments from four of these MDR donor lines were introgressed in the backgrounds of two multiple disease susceptible (MDS) lines (Lopez-Zuniga *et al.* 2019). Disease resistance QTL were identified in these populations, some associated with resistance to two or three diseases (Lopez-Zuniga 2016; Lopez-Zuniga *et al.* 2019).

The use of CSSL mapping populations allows for the comparison of QTL in a common background and facilitates follow up studies of specific alleles of interest. Since a particular donor allele is present in only a few lines in a CSSL population, however, effect estimates are prone to greater inaccuracy than in biparental mapping populations, where contrasting alleles are present at approximately equal frequencies. Also, since the lines are nearly completely homozygous, it is not possible to estimate dominance effects (Kaeppler 1997; Keurentjes *et al.* 2007; Jamann *et al.* 2015).

The goal of this study was to independently test the significance, and more precisely estimate the additive and dominance effects of some of the previously-identified disease resistance QTL (Lopez-Zuniga 2016), especially those associated with MDR. Twelve F_2:3_ populations were made from crosses between CSSL with the strongest resistance across the three diseases and their recurrent parent, H100. The resulting populations were assessed for each of the three diseases in replicated trials.

## Materials and methods

### Populations

As part of a previous study (Lopez-Zuniga *et al.* 2019), four chromosome segment substitution populations (BC_3_F_4:5_ lines) were created by crossing four multiple disease resistance maize (*Zea mays*) lines, Ki3, NC262, NC304, and NC344 as donors, with the disease susceptible lines H100 and Oh7B, as the recurrent parent. An identification code was ascribed to each line, starting with the prefix DRIL (for “Disease Resistance Introgression Line”) followed by the population code followed by a specific line number. The line codes were: Ki3 = 3, NC262 = 5, NC304 = 6, NC344 = 7, H100 = 2, Oh7B = 8). In each case, the code number for the donor parent is first and the number for the recurrent parent is second, so, for instance, 32 and 52, meant Ki3/H100 and NC262/H100 populations respectively. The final populations were genotyped using Pioneer Illumina publicplex platform with 765 SNP markers (Jones *et al.* 2009) with 245-271 informative SNPs within each population (Lopez-Zuniga *et al.* 2019). All the populations were evaluated for SLB, NLB, and GLS in replicated trials in two locations.

**Figure 1:**
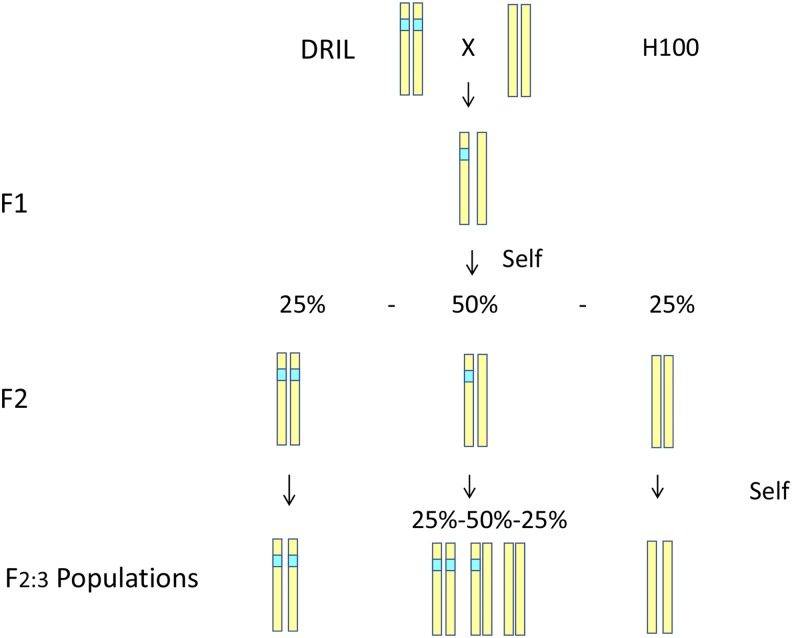
Scheme used to produce populations of F_2:3_ families and the QTL segregation within line.

For this study, twelve DRIL lines with H100 as the recurrent parent that showed strong resistance to all three diseases were chosen and crossed with H100 to generate F_2:3_ families (Figure 1, Table 1).

**Table 1:** F_2:3_ populations were derived from crosses between disease-resistant near-isogenic (DRIL) lines that had previously been identified as highly multiply disease resistant (Lopez-Zuniga 2016) and H100, the original susceptible recurrent parent used to construct the DRILs. Original donor parent indicates the original resistant parent used for the production of the DRIL lines (the original source of all the non-H100 alleles segregating in the F_2:3_ population). The number of F_2:3_ families created, and number of distinct introgressions previously associated with disease resistance segregating in each population are also presented

F_2:3_ Population name	Susceptible parent	DRIL parent	Original donor parent	Number of F_2:3_ families created	Number of introgressions
H100_DRIL_32.090	H100	DRIL_32.090	Ki3	70	2
H100_DRIL_32.095	H100	DRIL_32.095	Ki3	86	2
H100_DRIL_32.134	H100	DRIL_32.134	Ki3	63	2
H100_DRIL_32.191	H100	DRIL_32.191	Ki3	86	1
H100_DRIL_52.055	H100	DRIL_52.055	NC262	68	3
H100_DRIL_52.157	H100	DRIL_52.157	NC262	101	1
H100_DRIL_52.268	H100	DRIL_52.268	NC262	77	3
H100_DRIL_62.030	H100	DRIL_62.030	NC304	49	1
H100_DRIL_62.078	H100	DRIL_62.078	NC304	87	2
H100_DRIL_62.156	H100	DRIL_62.156	NC304	63	2
H100_DRIL_72.061	H100	DRIL_72.061	NC344	79	2
H100_DRIL_72.232	H100	DRIL_72.232	NC344	63	1

### Experimental design

Twelve populations, varying from 49 to 101 F_2:3_ families (Table 1) were tested in replicated trials for each of the diseases: SLB, NLB, and GLS. Each experiment had an augmented compete block design, with the population as block. The recurrent susceptible parent H100 was included at a random location within each sub-block of 21 plots, and each MDR parent was planted once in its respective population.

During the summer of 2017, two complete replications of 1029 plots each were planted for the evaluation of SLB in Central Crops Research Station (CCRS) Clayton NC; two complete replications were planted in Blacksburg VA, College Farm Research Station, for the evaluation of GLS; and one incomplete replication consisting of 700 plots, was planted in Andrews NC for the evaluation of GLS. During summer 2018, two complete replications were planted in CCRS, Clayton NC, for the evaluation of NLB.

### Inoculation preparation and inoculum procedure

The inoculum was prepared as previously described (Sermons and Balint-Kurti 2018). Briefly, sorghum kernels were soaked in water for 3-4 days, autoclaved for one hour, allowed to cool overnight and inoculated with either *C. heterostrophus*, *S. turcica*, or *C. zeae-maydis*. The fungus was grown at room temperature (23-25°) for at least 10 days until the sorghum was colonized. The fungus-infested sorghum was air-dried and stored at 4°. 30-40 day old maize plants were inoculated by adding 6-10 infested sorghum kernels into the whorl of each maize plant.

### Genotyping

Tips of leaves were collected from 5 adult plants per F_2:3_ family, bulked, and lyophilized. The samples were sent to Agriplex Genomics for DNA extraction and genotyping. For each population, only markers known to be segregating and that were associated with resistance to one or more diseases in the previous study (Lopez-Zuniga *et al.* 2019) were genotyped. Primers were designed based on the context sequence of the SNPs reported by Lopez-Zuniga (2016) using the B73 reference genome version 3. (Table S1). All the genotypic data used are available in File S1. Genetic map positions are based on the IBM4 genetic map (Fu *et al.* 2006).

### Phenotypic evaluation of SLB

The experiment was planted in a single field, with 8 seeds per plot, in 1.8-m single rows with 0.9-m row width. Inoculations of SLB were performed 40 days after planting, using *C. heterostrophus* isolates, including 2-16Bm and Hm540 (Carson 1998). Visual scores were taken three times at 10 days interval, starting 77 days after planting, when the plants were in the developmental stage R2. The scoring used a scale of 1 to 9, where 1 is dead and 9 is immune (Sermons and Balint-Kurti 2018). Each plot had one score given at each evaluation. If the plot was segregating it was given a score that represented the average disease severity of the plot. For each plot in both replications, days to anthesis (DTA) was recorded when half the plants in a plot were shedding pollen. File S2 includes all the SLB scoring data.

### Phenotypic evaluation of NLB

All field evaluations of NLB were performed during summer growing season of 2018, with two replicates grown at CCRS in Clayton, NC. The experiment was planted in the same fashion as the SLB trial. Inoculations of NLB were performed 26 days after planting, using several *Exserohilum turicum* isolates (race 0, race 1, race 2,3 and race 2,3,N). Visual scores were taken three times at six to eleven days interval, starting 64 days after planting. NLB was scored using the percentage of diseased leaf area, from 0 to 100. A single score was ascribed to each plot at each evaluation. If the plot was segregating it was given a score that represented the average disease severity of the plot. The disease scores were converted to fit the same scale of GLS and SLB (1 to 9). For each plot in both replications, days to anthesis (DTA) was recorded when half the plants in a plot were shedding pollen. File S3 includes all the NLB scoring data.

### Phenotypic evaluation of GLS

All field evaluations of GLS were performed during the summer growing season of 2017, with two replicates at College Farm Research Station in Blacksburg, VA and an incomplete replicate in Andrews, NC. Trials were planted in 4-m single rows with a 1-m row width using 15 seeds per plot, in both locations. Inoculation was performed in the field in Virginia 30 days after planting, using a mixture of several *Cercospora maydis* inoculum isolates. No artificial inoculation was done in Andrews since the field contained infected plant debris from previous years that provided a high disease pressure. Visual scores were taken twice at each location using a scale of 1 to 9, where 1 is dead and 9 is immune A single score was ascribed to each plot at each evaluation. If the plot was segregating it was given a score that represented the average disease severity of the plot. Scores were recorded with the Field Book application (Rife and Poland 2014) File S4 includes all the GLS scoring data.

### Statistical analysis

#### Phenotypic data:

Exploratory phenotypic data analysis was performed using Statistical Analysis System (SAS) v9.4 software (SAS Institute Inc., Cary, NC), Tableau v9.1 and R (R core development team 2015). We did not find any outlying data points that warranted removal. Heat maps were produced to visualize any disease severity spatial pattern in the field and box plots were created to check the severity of disease in the F_2:3_ lines in comparison to the susceptible and resistant checks. Correlation coefficients between disease scores of lines measured in different replications or on different diseases were calculated.

For each disease trial, using two to three disease scores collected at different time points, the standardized area under disease progress curve (sAUDPC) was calculated for each plot by taking the average value of two consecutive ratings and multiplying by the number of days between the ratings. Values were then summed over all the intervals and then adjusted by dividing by the number of days between evaluations, so that the sAUDPC scores are on a similar one to nine scale as the initial ratings (Campbell and Madden 1990).

Statistical analysis was performed using the MIXED procedure in SAS. To adjust the least square means (LSMeans) for field effects, range and column of the field position for each plot were added to the model as random effects. The mixed linear model used to analyze data from the SLB trials was

$$y_{ijkmn}=\mu +L_{i\;}+\;D_{j}\;+\;R_{k}+G_{m}\;+\;C_{n}+\;\varepsilon_{ijknm}\;\;$$

where y is the response variable SLB sAUDPC, *L_i_* is the fixed effect of the *i*^th^ line, *D_j_* is the fixed effect of number *j^th^* of days to anthesis (DTA), *R_k_* is the random effect of the *k*^th^ replicate, *G_m_* is the random effect of the *m*^th^ range, *C_n_* is the random effect of the *n*^th^ column. A similar model was used to analyze data from the NLB trial:

$$y_{ijkmn}=\mu +L_{i\;}+\;D_{j}\;+\;R_{k}+G{\left(R\right)}_{m(k)}\;+\;C{\left(R\right)}_{n(k)}+\;\varepsilon_{ijknm}\;\;$$

the response variable y was NLB AUDPC, *L_i_* is the fixed effect of the *i*^th^ line, *D_j_* is the fixed effect of number *j^th^* of days to anthesis (DTA), *R_k_* is the random effect of the *k*^th^ replicate, *G(R)_m(k)_* is the random effect of the *m*^th^ range nested in *k*^th^ replicate, *C(R)_n(k)_* is the random effect of the *n*^th^ column nested in *k*^th^ replicate. To analyze the data from the GLS field trial, the model used was

$$y_{ijkmn}=\mu +L_{i\;}+\;O_{j}\;+\;R{\left(O\right)}_{k(j)}+G{\left(O\right)}_{m(j)}\;+\;C{\left(O\right)}_{n(j)}+\;\varepsilon_{ijknm}$$

where **y** is the response variable GLS AUDPC, *L_i_* is the fixed effect of the *i*^th^ line, *O* is the random effect of *j^th^* location, *R(O)*_k(j)_ is the random effect of *k*^th^ replicate nested in *j^th^* location, *G(O)_m(j)_* is the random effect of *m^ith^* range nested in *j^th^* location, *C(O)* is the random effect of *n^th^* column nested in *j^th^* location. LSMeans for each line and disease were calculated from the model.

Heritability on a plot and entry-mean basis for each disease were calculated as described previously (Holland *et al.* 2003) using the variance components described above and a harmonic mean for the number of replicates per entry. We used combined data from all populations to estimate heritability.

### Genotypic data

A Chi-square test was performed for each marker within each population to assess if the segregation was significantly different from the expected 1:2:1. Markers whose segregation was significantly different to expectation were removed from the study.

Each marker was tested separately by multiple regression of F_2:3_ family LSMeans as observations on coefficients representing the additive and dominance effects at the marker using SAS PROC MIXED. Additive coefficients represented the number of resistant parent alleles (0, 1, or 2) at the locus in the F_2_ founder plant of each family. Dominance coefficients were 0 for both homozygous classes and 1 for heterozygotes. Marker additive effects were estimated directly from the additive regression coefficient; dominance effects were estimated as twice the dominance regression coefficient because only half of the individuals in the segregating lines are expected to be heterozygous. Type III F-tests of additive and dominance regression coefficients were used to declare significant marker effects at α=0.05. If more than one marker was significant in the same bin and less than 20 cM apart, the marker that had the smallest P-value, or had a significant effect for the greatest number of diseases, was chosen to represent the effect of that QTL (Table 2, Table S2). In cases where the population was segregating for more than one unlinked marker, marker interactions were tested by fitting the two markers and their interaction in the model using PROC GLM to verify epistasis. If there was no interaction, a third model was run with PROC GLM, fitting the main effects of the two markers as fixed effects and the disease LSMeans as the response to obtain simultaneous estimates of the two marker effects.

**Table 2:** Results from selected markers to represent each introgression. Multiple disease resistant donor line (Donor), code name of NIL parent carrying target introgression and crossed to H100 to form F_2:3_ families for validation, marker, chromosome (Chr) and genetic bin, genetic position (cM) based on the IBM4 genetic map, trait and additive effect estimate ($\hat{a}$) previously associated with the marker (Lopez-Zuniga 2016), disease, additive effect ($\hat{a}$), dominance effect ($\hat{d}$), and trait(s) for which QTL effect was validated by this study. Non-significant dominance effect are denoted as NS. All reported marker effects are significant at *P* = 0.05

					QTL effect detected in previous study		Significant QTL effects detected in current study			
Donor	DRIL parent code	Marker	Chr/Bin	cM	Trait	$\hat{a}$	Disease	$\hat{a}$	$\hat{d}$	Trait(s) with validated QTL effect
Ki3	DRIL32.090	PHM3457-6	2.05	96.4	MDR	0.39	GLS	0.18	NS	MDR
							NLB	0.07	NS	
	DRIL32.090	PZA00379-2	8.03	59.5	MDR, SLB	0.27, 0.28	SLB	0.16	NS	MDR, SLB
							NLB	0.10	NS	
	DRIL32.095	PZA01886-1	9.04	114.7	MDR, SLB	0.50, 0.29	SLB	0.30	NS	SLB
	DRIL32.134	PZA00485-2	2.05	99.1			GLS	0.28	NS	
	DRIL32.134	PHM4757-14	8.03	89.8	MDR, GLS, NLB	0.24, 0.16, -2.73	NLB	0.13	NS	NLB
	DRIL32.191	PHM4495-14	9.03	59.0	MDR, SLB	0.36, 0.25	NLB	0.05	NS	
NC262	DRIL52.055	PZA03577-1	2.07	195.7	MDR	0.36	SLB	−0.19	NS	
	DRIL52.055	PZA00060-2	9.04	114.5	MDR	0.33	SLB	0.18	NS	MDR
							NLB	0.03	NS	
	DRIL52.268	PZA03577-1	2.07	195.7	MDR	0.36	GLS	0.13	NS	
	DRIL52.268	PHM4757-14	8.03	98.8	GLS	0.35	GLS	−0.11	NS	
NC304	DRIL62.030	PHM13420-11	3.04	92.1	MDR, GLS	0.54, 0.17	GLS	0.32	NS	GLS
NC304	DRIL62.078	PHM9635-30	4.05	96.0	GLS	0.17	GLS	0.26	NS	GLS
							NLB	0.05	NS	
NC304	DRIL62.078	PZA02209-2	5.04	124.0	MDR	0.25	GLS	0.15	NS	MDR
							SLB	0.14	NS	
							NLB	0.06	NS	
NC304	DRIL62.156	PHM13420-11	3.04	92.1	MDR, GLS	0.54, 0.17	GLS	0.27	NS	MDR, GLS
						SLB	0.43	NS		
						NLB	0.08	0.14		
NC344	DRIL72.061	PHM14412-4	2.05	127.4	MDR, GLS	0.30, 0.16	GLS	0.27	0.54	GLS
NC344	DRIL72.061	PZA00667-1	3.04	96.7	MDR	0.35	SLB	0.21	NS	MDR
							NLB	0.07	NS	
NC344	DRIL72.232	PHM4586-12	2.05	79.3	GLS	0.14	NLB	−0.07	NS	

### Data availability

All phenotypic and genotypic data used in this study are included in Files S1-4. In many cases very little seed remains of F_2:3_ families used. However F_2_ populations are available upon request. The original DRILs are available at the maize genetic stock center (https://maizegdb.org/data_center/stock?id=9039691).

All phenotypic and genotypic data used in this study are available in the supplementary files. Further information is available from the corresponding authors if required. Supplemental material available at FigShare: https://doi.org/10.25387/g3.7887698.

## Results and Discussion

Twelve F_2:3_ populations developed from crosses between 12 selected disease resistant lines and the susceptible parent H100 were created (Figure 1, Table 1). These populations were evaluated in replicated trials for SLB, NLB and GLS resistance. Correlations between replications were all highly significant. Heritabilities on a plot-basis were 0.45, 0.29, and 0.36 and on an entry-mean basis were 0.57, 0.49, and 0.52 for SLB, GLS, and NLB, respectively. Pairwise phenotypic correlations across all the populations for resistance to the three diseases were moderate but highly significant ranging from *r* = 0.2 to 0.3 (*P*-value <0.01). The pairwise disease resistance correlations within individual populations were often but not always significant (Table S3). Correlation and heritability estimates were relatively moderate due partly to the nature of these populations. Each population was only segregating at a small number of loci and therefore captured less genetic variation than typically seen in RIL populations or association panels, for which a large proportion of the genome is variable.

The fixed effects of line and DTA (when DTA data were available) were significant for all diseases. For all field trials, range and column field effects were significant but replicate was never significant (Table S4). The only disease that had field trials in different locations was GLS and the effect of location was not significant.

Each of the 12 DRILs selected as the parents of the 12 F_2:3_ populations carried multiple introgressions. Of these, between one and three introgressions had been previously associated with resistance to at least one disease or the MDR composite score in our previous analyses (Lopez-Zuniga 2016). We followed the segregation of all of these previously associated introgressions for all subsequent analyses, but we did not follow the segregation of any introgressions that were segregating in the population that had not been associated with resistance to any disease in the original analysis.

We genotyped 28 markers (Table S1) to examine the segregation of 24 introgressions across the twelve F_2:3_ family populations (Table 1). Each population was segregating at one to five of these markers/introgressions. We assessed the association of each segregating marker with resistance to each disease regardless of which disease(s) it had been associated with in the previous study. In total, associations between 66 QTL-disease combinations (with some QTL represented by more than one marker) were tested. In cases in which more than one marker was used to follow the segregation of a single introgression, we selected the marker most strongly associated with disease resistance as the marker to represent the QTL in that introgression for further analyses (Table S2). The QTL was considered validated if it had the same directional allele effect on any of the diseases as was found previously (Lopez-Zuniga 2016). In cases where two markers representing different introgressions were both significant for a specific disease/population combination, both were incorporated in a final model to calculate effect sizes to account for any potential epistatic effects, though ultimately no epistatic effects were detected.

After selecting only one marker for each introgression, we were able to test 44 previously identified QTL, 20 associated with the MDR composite score and 24 with single disease resistance, several of which co-localize (Table S2). The effects of 16 out of these 44 previously identified QTL were validated. Table 2 summarizes the significant marker/disease resistance associations that were detected. An MDR QTL that was previously detected with the composite score (Lopez-Zuniga 2016) was considered validated when it was significant for more than one disease. In some cases, the same QTL was validated for MDR and single disease resistance. In the original analysis, MDR QTL were detected using an initial analysis based on a composite score (equally weighted index) derived from the average of the trait values of the three diseases (Lopez-Zuniga 2016). Subsequent to the development of the populations for this study, an alternative analysis of the original CSSL populations was performed using a composite statistic, *Md*, which accounts for covariance between the QTL test results for each trait (Lopez-Zuniga *et al.* 2019). The MDR loci that were targeted in the present study were identified based on the original MDR QTL analysis (Lopez-Zuniga 2016). Of the 20 MDR alleles tested over all the populations (Table S2), 18 were also identified as MDR alleles in the analysis with the *Md* statistic. Of the two alleles not detected using the *Md* statistic, (PHM3457-6/Ki3 and PZA03577-1/NC262) one (PHM3457-6/Ki3) was validated for MDR in this study (Table 2, Table S2).

Considering multiple and single diseases, we identified 33 marker-disease associations in this study. Since 16 of these were validations of previously identified QTL, that means that 17 were not identified in the previous analysis of the CSSL populations (Lopez-Zuniga *et al.* 2019). For example, marker PHM4586-12 in population H100_DRIL72.232 was previously associated with GLS but in the current study was significant for NLB. Marker PHM4495-14 in population H100_DRIL32.191 was previously associated with SLB but in the present study, it was significant for NLB.

Dominance was observed only in two cases out of the 26 instances where we observed a significant marker-trait association: marker PHM13420-11 in population H100_DRIL62.156 and marker PHM14412-4 in population H100_DRIL72.061 (Table 2). In both cases the allele conferring resistance was dominant. This relative rarity of dominance effects may be partially due to the low power with which we are able to detect them; only half the plants in an F_2:3_ family that is derived from a heterozygous parent are themselves heterozygous, so only half the full dominance effect can be observed and thus dominance is harder to observe than if we were comparing fully homozygous and fully heterozygous families. It may also be the case that QDR alleles tend not to show dominance effects. It is hard to determine from the literature whether this is the case as most studies have been conducted with RIL populations from which no dominance effects can be calculated

While alleles from the MDR parent conferred resistance as expected in most cases, in three of 26 cases, alleles from the donor MDR parent were associated with disease susceptibility. The NC262 allele at marker 2PZA03577-1 carried a susceptibility allele for SLB in population H100_DRIL52.055, the NC262 allele at marker PHM4757-14 conferred susceptibility to GLS in population H100_DRIL52.268 and the NC344 allele at marker PHM4586-12 conferred susceptibility to NLB in population H100_DRIL72.232 (Table 2). It is not surprising that a few resistance alleles derived from the susceptible parent. This phenomenon has been observed a number of times in our previous studies (*e.g.*, Balint-Kurti *et al.* 2007).

Possible reasons why fewer than half the QTL were validated are associated with the design of both the original study and this validation study. The low minor allele frequency in the original CSSL mapping populations, an inherent feature of these populations, might have caused inaccuracies since each minor allele was only sampled a handful of times in each population. Also, since the genotyping of the original populations was not dense (209-271 markers in each population) it is possible that some introgressions that affected disease resistance were not accounted for, which may have led to greater errors in the analysis. In the current study, the number of F_2:3_ families used in each population was between 49 and 101 (Table 1), a relatively low number of families which may not have provided enough power to detect QTL of modest effect. We noted previously (Lopez-Zuniga *et al.* 2019) that the detection of an MDR locus could be driven by a strong effect on one disease and little or no effect on the others. In this study, we deliberately took a more stringent approach, only declaring an MDR QTL if it had a significant effect on more than one individual disease. In several cases, a marker previously associated with MDR was associated with resistance to only one disease and so by our criteria, it was not considered to be validated. For example, marker PHM14412-4 on population H100_DRIL72.061 was associated with GLS and MDR previously but was validated only for GLS (Table 2). In cases like this, it is likely that the original MDR QTL might have been identified due to strong effects on one disease (GLS) in this case rather than to moderate effects on multiple diseases. In fact, most of the QTL identified using the *Md* statistic had effects strongly skewed toward one disease (Lopez-Zuniga *et al.* 2019). Conversely, the new marker/disease associations identified in this study likely occurred because the F_2:3_ populations had more power to test associations due to a higher representation of the minor allele.

In some cases, the same allele was significant for the same disease in different populations. Marker PHM13420-11 associated with an allele from NC304 was validated for GLS in population H100_DRIL62.030 and for GLS and MDR in population H100_DRIL62.156 (Table 2). In these cases, the fact that it was validated in distinct F_2:3_ populations increases our confidence in the association of this marker and GLS resistance. On the other hand, there were instances where the same allele was segregating in two populations but the results from single marker analysis differed. For example, Marker PHM3457-6 was significant for all three diseases in H100_DRIL32.090 and only for GLS in H100_DRIL32.134 (Table 2). This could have occurred due to the type of experimental errors described above or possibly the marker may have been linked to the causal gene in one population but may have become unlinked in another due to a recombination event occurring during the development of the parental DRIL.

The main goal of this study was to validate and characterize robust MDR alleles. Of the six alleles validated for MDR, two were associated with resistance to all three diseases; in population H100_DRIL62.078, marker PZA02209-2, bin 5.04 and in population H100_DRIL62.156, marker PHM13420-11, bin 3.04. In both cases the resistance alleles derived from the maize line NC304. Both loci have been previously identified as QTL for all three diseases (Table 3). However, in none of these cases was the same allele associated with resistance to all three diseases. Previous meta-analyses of maize QTL have also identified bin 3.04 as a disease resistance QTL hotspot (McMullen and Simcox 1995; Wisser *et al.* 2006; Ali *et al.* 2013).

**Table 3:** Summary of QTL for SLB, NLB and GLS identified in previous studies in bins 3.04 and 5.04. Table includes BIN, marker, chromosome (Chr), population type (pop), disease (Dis), mapping method (Method) and reference

BIN	Marker	Pop	Dis	Method	Reference
3.03/3.04	asg48-phi036	B73*Mo17 (RILs)	SLB	CIM	(Carson *et al.* 2004)
3.04	UMC10	F2:3, ADENT*B73rhm	GLS	SIM	(Bubeck *et al.* 1993)
3.04	PIO200508	F2:3, ADENT*B73rhm	GLS	SIM	(Bubeck *et al.* 1993)
3.04	BNL10.24	F2:3, ADENT*B73rhm	GLS	SIM	(Bubeck *et al.* 1993)
3.04	us41	Propietary F2	GLS	CIM	(Lehmensiek *et al.* 2001)
3.04	PHM4621.57	NAM	NLB	GWAS	(Poland *et al.* 2011; Li *et al.* 2018)
3.04	phi036-bnlg602	NC300*B104 (RILs)	SLB	MIM	(Balint-Kurti and Carson 2006)
3.04	PZA02077	NILs, Teosinte*B73	SLB	LA	(Lennon *et al.* 2014)
3.04	PZA00828	NILs, Teosinte*B73	SLB	LA	(Lennon *et al.* 2014)
3.04	PHM4145_18	B73*CML254, B97*CML254/Ki14 (RILs)	SLB	JL	(Negeri *et al.* 2011)
3.04	umc2275-umc2008	T14*T4 F2:3	SLB	CIM	(Pengfei *et al.* 2011)
3.04	umc2275-umc2008	T14*T4 F2:3	SLB	CIM	(Pengfei *et al.* 2011)
3.04	npi446-umc2000	B73*Mo17 (AIRIL)	SLB	CIM	(Balint-Kurti *et al.* 2007)
3.04	mmp69-umc1920	B73*Mo17 (AIRIL)	SLB	CIM	(Balint-Kurti *et al.* 2007)
3.04	PHM4204.69-PHM2343.25	NAM	SLB	GWAS	(Kump *et al.* 2011; Li *et al.* 2018)
3.04	asg48-phi036	B73*Mo17 (RILs)	SLB	CIM	(Balint-Kurti and Carson 2006)
3.04/3.05	umc010-umc 389b	F2:3 Lo951*CML202	NLB	CIM	(Welz *et al.* 1999a)
5.03/5.04	umc001-bnl5.40	F2:3 Lo951*CML202	NLB	CIM	(Welz *et al.* 1999a)
5.03-5.04	umc1171-bnlg1046	BC1F4, Y32*Q11	GLS	LA	(Zhang *et al.* 2012)
5.04	bnlg150	Propietary F2	GLS	CIM	(Lehmensiek *et al.* 2001)
5.04	UMC43 UMC40	BC1S5, FR1141*O61	GLS	CIM	(Clements *et al.* 2000)
5.04	UMC40 BNL7.71	BC1S5, FR1141*O61	GLS	CIM	(Clements *et al.* 2000)
5.04	ASG71 CSU440	BC1S5, FR1141*O61	GLS	CIM	(Clements *et al.* 2000)
5.04	BNL6.22-UMC51	HighLand*LowLand	NLB	CIM	(Jiang *et al.* 1999)
5.04	PHM532.23	NAM	NLB	GWAS	(Poland *et al.* 2011; Li *et al.* 2018)
5.04	csu36a−bnl7.71	F3 D32*D145	NLB	CIM	(Welz *et al.* 1999b)
5.04	BNL5.7I-UMC51	B52*Mo17 F2:3	NLB	SIM	(Freymark *et al.* 1993)
5.04	bnl5.40-npi461	F2:3 Lo951*CML202	NLB	CIM	(Welz *et al.* 1999a)
5.04	PZA03049.24-PZB01017.1	NAM	SLB	GWAS	(Kump *et al.* 2011; Li *et al.* 2018)
5.04/5.05	umc068-bnl5.24	F2:3 Lo951*CML202	NLB	CIM	(Welz *et al.* 1999a)

Acronyms: CIM: composite interval mapping; GWAS: genome wide association study; JL: joint-linkage analysis; LA: Linkage analysis; MIM: multiple interval mapping; SIM: simple interval mapping. Adapted from (Lopez-Zuniga 2016).

This study demonstrates the importance of independently evaluating QTL effects following their initial identification and before deploying them in a breeding program. In many cases where validation has been carried out (*e.g.*, Lennon *et al.* 2016, 2017), the validation rate was substantially higher than that reported here. It is nevertheless quite possible that QTL identified in single studies using a handful of environments may not be robust. Disease QTL, and QTL in general, often show environment-specific effects (*e.g.*, Bubeck *et al.* 1993) and genetic background and epistasis are also common factors affecting QTL effects (Holland 2007; Kump *et al.* 2010). This study further confirms the existence of QTL associated with multiple diseases that could be exploited in breeding programs. Future studies could fine map and investigate genes behind the MDR QTL to better understand the mechanisms that plants use to resistance against pathogens.
